# Analysis of Delayed Bleeding after Endoscopic Submucosal Dissection for Gastric Epithelial Neoplasms

**DOI:** 10.1155/2012/875323

**Published:** 2012-02-08

**Authors:** Shinichi Mukai, Songde Cho, Takahiro Kotachi, Akinori Shimizu, Genta Matuura, Michihiro Nonaka, Toshihide Hamada, Ken Hirata, Toshio Nakanishi

**Affiliations:** Department of Internal Medicine, Miyoshi Central Hospital, 531 Higashisakeya-chou, Miyoshi, Hiroshima 728-8502, Japan

## Abstract

*Aim*. Delayed bleeding after endoscopic submucosal dissection (ESD) for gastric epithelial neoplasms is a major complication. We investigated factors related to post-ESD bleeding to identify preventive measures. *Methods*. The study included 161 gastric epithelial neoplasms in 142 patients from June 2007 to September 2010. Post-ESD bleeding was defined as an ulcer with active bleeding or apparent exposed vessels diagnosed by an emergency endoscopy or a planned follow-up endoscopy. We analyzed associations between bleeding and the following factors: age, sex, morphology, pathology, tumor depth, ulcer presence/absence, location, size of the resected lesion, duration of the procedure, the number of times bleeding occurred during ESD, and the use of anticoagulants and/or antiplatelet drugs. Subsequently, we examined characteristics of bleeding cases. *Results*. Post-ESD bleeding occurred in 21 lesions. Univariate analysis of these cases showed that ulcer presence/absence (*P* < 0.001), middle or lower third lesions (*P* = 0.036), circumference (*P* = 0.014), and a post-ESD ulcer with an extended lesser curve (*P* = 0.009) were significant predictors of bleeding. Multivariate analysis showed that ulcer presence/absence (OR 9.73, 95% CI 2.28–41.53) was the only significant predictor. *Conclusion*. Ulcer presence/absence was considered the most significant predictor of post-ESD bleeding.

## 1. Introduction

Endoscopic submucosal dissection (ESD) for gastric adenomas and early gastric cancers enables en-block resection even for large or ulcerative lesions [1, 2]. However, ESD is technically demanding and is a lengthy procedure. Furthermore, ESD can cause additional complications such as perforation and bleeding compared with endoscopic mucosal resection (EMR) [1–3]. Recently, ESD has been widely performed in general hospitals, and the indications for ESD have been expanded according to new criteria [4]. Accordingly, measures to address major complications such as perforation and bleeding have become increasingly important. Although the frequency of complications has been decreasing with improvements in technique and instrumentation for ESD, post-ESD bleeding occurs in approximately 5% of cases [3, 5–7]. When post-ESD bleeding occurs, patients may suffer from hematemesis, hemorrhagic shock, and other complications, which may require emergency endoscopy and prolonged fasting. Medical staff are also impacted by the need to perform emergency treatment. Several previous studies have reported investigations of risk factors for post-ESD bleeding [6, 8–12]. However, there is currently no consensus with respect to these risk factors. Therefore, we investigated factors related to post-ESD bleeding in our patients to identify preventive measures.

## 2. Materials and Methods

### 2.1. Subjects

A total of 161 gastric epithelial neoplasms in 142 patients (125 early gastric cancers and 36 gastric adenomas) were consecutively treated with ESD in our hospital from June 2007 to September 2010. ESD was determined to be indicated for gastric epithelial neoplasms according to the criteria of extended indication of ESD for gastric cancer in the institute. The criteria are the following: (i) any tumor size if it was a differentiated adenocarcinoma (intestinal type) without ulceration or submucosal invasion, (ii) tumor size equal to or less than 3 cm if tumor with ulceration was suspected and it was a differentiated adenocarcinoma without submucosal invasion, (iii) tumor size equal to or less than 2 cm if tumor was a undifferentiated adenocarcinoma (diffuse type) without ulceration or submucosal invasion. All patients provided informed consent before undergoing the procedures, and patient anonymity was preserved throughout the study. Furthermore, because this was a retrospective study with patient anonymity, we have not taken study approval from our institutional review board.

### 2.2. ESD Procedures

ESDs were performed according to a standard protocol [1, 2]. An operator and an assistant performed the procedure using a video endoscope (GIF-Q260J; Olympus, Tokyo, Japan) and various instruments for ESD. The operators included two experts (with an experience of more than 50 cases) and two beginners. When a beginner performed the procedure as an operator, an expert provided complete support as an assistant and replaced the operator as needed. After marking the lesion margins using an APC probe (VIO 300D; Erbe, Tubingen, Germany), a submucosal injection was performed to lift the lesion. A mixture of 10% glycerin and hyaluronic acid containing 0.5% indigocarmine and 0.1% epinephrine was used as the injection fluid. A circumferential incision was performed using an instrument such as needle knife, insulated-tip knife (KD-610L, 611L; Olympus, Tokyo, Japan), or flush knife (DK2618JN20; Fujinon, Tokyo, Japan), according to the operator's preference. Continuous submucosal dissection of the lesion was performed. Hemostatic forceps (FD-410LR; Olympus) were used to control bleeding during the procedure. When ESD was completed, coagulation of visible vessels in the resection area was also performed using hemostatic forceps to prevent delayed bleeding. A proton pump inhibitor (PPI), 20 mg omeprazole, was administered intravenously twice daily from the day of ESD to the day before beginning a soft diet. Oral administration of PPIs, 20 mg rabeprazole was continued from the day on which meals were begun for approximately 2 months. A second-look endoscopy was routinely performed the next day or a few days after ESD before beginning meals, and a third-look endoscopy was generally performed 8 days after ESD before discharge, primarily to prevent delayed bleeding. If bleeding or visible vessels that had the potential for bleeding were detected on the ulcer, preventive treatment was performed using hemostatic forceps or clipping with hemostatic clips (HX-610-135; Olympus). If bleeding with hematemesis or melena occurred, emergency endoscopic hemostasis using hemostatic forceps, clipping, injection of hypertonic saline epinephrine (HSE), and other necessary treatments were performed. Generally, if adverse events, bleeding, perforation, pneumonia, or other complications did not occur, patients were discharged within 10 days after ESD. A follow-up endoscopy was performed 2 months after ESD to confirm healing of the ulcer and to detect new lesions.

### 2.3. Data Analysis

**Figure 1:**
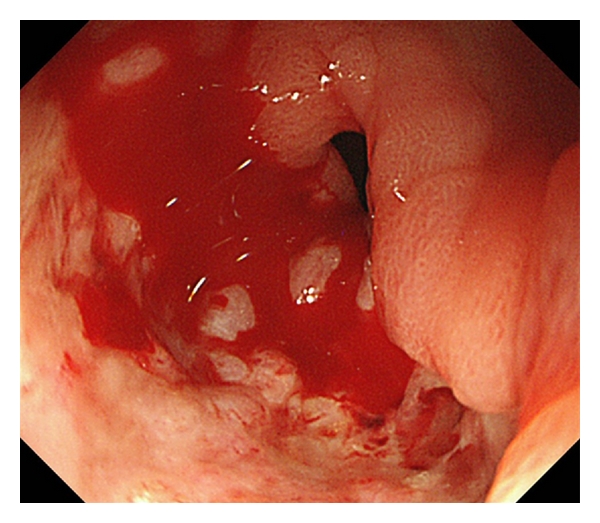
Endoscopic appearance of post-ESD ulcer with active bleeding.

**Figure 2:**
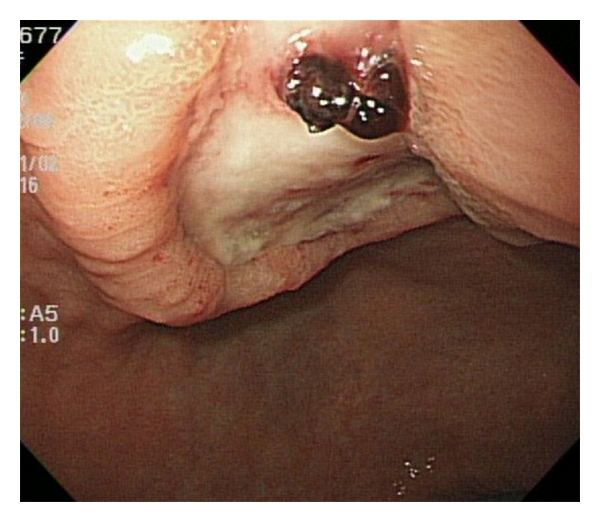
Endoscopic appearance of post-ESD ulcer with apparent exposed vessels.

Post-ESD bleeding was defined as active bleeding from a post-ESD ulcer diagnosed by an emergency endoscopy or a planned follow-up endoscopy (Figure 1). In addition, we defined occult bleeding cases with a high risk of bleeding as post-ESD bleeding. They were cases with a post-ESD ulcer with active bleeding after washing with water or a water jet (Olympus) or apparent exposed vessels when a planned follow-up endoscopy was carried out (Figure 2).

To investigate factors influencing post-ESD bleeding, we analyzed the following factors and compared the groups with post-ESD bleeding to the control group: age, sex, morphology (0–I, IIa, IIc, IIa + IIc, or III), pathology (intestinal type, diffuse type, or adenoma), tumor depth (mucosal tumor or submucosal tumor), ulcer findings (endoscopically or pathologically present or absent), tumor location in terms of the major axis (upper third (U), middle third (M), or lower third (L)), the circumference (anterior wall, posterior wall, lesser curve, or greater curve), size of the resected lesion (maximum diameter, mm), duration of the procedure (min), the number of times bleeding occurred during ESD (represented by the number of times hemostatic forceps were used), and whether anticoagulants and/or antiplatelet drugs were used. Subsequently, we classified the post-ESD ulcers into two groups, ulcers with an extended lesser curve and those without. We carried out the data analysis for cases with post-ESD bleeding versus the control cases.

Subsequently, we analyzed several characteristics of the cases with post-ESD bleeding, including the timing, region, and nature of the bleeding. Timing was classified into early phase (up to 2 days after ESD) and delayed phase (3 or more days after ESD). The region was classified in terms of the major axis (upper third, middle third, or lower third), the circumference (anterior wall, posterior wall, lesser curve, or greater curve), and location at the ulcer (ulcer margin or ulcer center). The ulcer margin was defined as bleeding vessels in the area adjacent to the ulcer edge. The ulcer center was defined as the ulcer floor area other than the ulcer margin. The nature of the bleeding was classified into four types (spouting, gushing, exposed vessels, or oozing). Furthermore, we analyzed the following symptoms and treatments for the cases: hematemesis, anemia (decline in Hb > 2 g/dL), hemorrhagic shock (systolic blood pressure < 90 mm Hg), and blood transfusion.

### 2.4. Statistical Analysis

Univariate and multivariate analyses were performed. Differences in the means of continuous data, such as age, size of the resected lesion, duration of the procedure, and the number of episodes of bleeding, were compared using Student's *t-*test for a normally distributed population; otherwise, Mann-Whitney *U* test was used. Categorical data, such as sex, morphology, pathology, tumor depth, ulcer presence/absence, tumor location, and whether anticoagulants and/or antiplatelet drugs were used, were compared using a *χ* ^2^ test and Fisher's exact test. Variables with a *P* value < 0.2 in the univariate analysis were included in a forward, stepwise multiple logistic regression model. *P* values < 0.05 were considered statistically significant and all tests were two sided. The analyses were performed using Ekuseru-Toukei 2010 (Social Survey Research Information Co. Ltd., Tokyo, Japan).

## 3. Results

Baseline data for the clinical characteristics of the 161 gastric lesions in 142 patients are displayed in Table 1. Post-ESD bleeding occurred in 21 (13.0%) of the 161 lesions, 9 active bleeding, and 12 occult bleeding cases. Perforation occurred in 2 patients in ESD, they were treated by endoscopic clipping and did not required surgical treatments.

Univariate analysis showed no significant differences between the 21 bleeding cases and the control cases in age, sex, morphology, pathology, tumor depth, size of the resected lesion, duration of the procedure, the number of episodes of bleeding, or the use of anticoagulants and/or antiplatelet drugs. Ulcer presence/absence (*P* < 0.001), location (upper versus middle or lower, *P* = 0.036), circumference (*P* = 0.014), and a post-ESD ulcer with an extended lesser curve (*P* = 0.009) were significantly different between the bleeding and control groups and were considered predictors for post-ESD bleeding (Table 2). The subsequent multivariate analysis showed that ulcer presence/absence (OR 9.73, 95% CI 2.28–41.53) was the only statistically significant predictor of post-ESD bleeding (Table 3).

We then investigated the 21 cases with post-ESD bleeding in detail. With respect to the timing of bleeding, 15 cases occurred in the early phase and 6 in the delayed phase. Although the majority of the cases occurred relatively early (within 2 days), some cases occurred about a week after ESD (3 cases at 8 days). Bleeding at the ulcer occurred at the ulcer margin in 9 cases and in the center of the ulcer in 12 cases. Bleeding findings, including 2 spouting, 8 gushing, 5 exposed vessels, and 6 oozings were observed. 6 cases had hematemesis, 4 had anemia, 4 had hemorrhagic shock, and a blood transfusion was performed in 2 cases (Table 4). All the cases of post-ESD bleeding were treated using endoscopic procedures and did not require surgical intervention.

## 4. Discussion

In the present study, the presence of ulcers was the only predictor for post-ESD bleeding with a significant difference. Location (middle- or lower-third region) and a post-ESD ulcer with an extended lesser curve were also considered predictors for post-ESD bleeding.

Post-ESD bleeding is a major complication of ESD along with perforation, and several previous studies have reported investigations of risk factors for post-ESD bleeding [6–12].

Previous studies have reported that the size of the resected tumor was the only significant risk factor for post-ESD bleeding [8, 9]. In our study, although the univariate analysis showed no significant difference between the post-ESD bleeding cases and the control cases in the size of the resected tumor, there was a tendency toward larger size in the bleeding cases. If a larger number of cases had been investigated, the size of the resected tumor might have been a significant predictor of post-ESD bleeding.

A previous study reported that pathology was a significant risk factor for post-ESD bleeding [10]. In our study, the analysis showed no significant differences related to pathology.

There is currently no consensus in the literature with respect to the effect of the location of the major axis (upper, middle, and lower). Several previous studies have not reported any significant findings for lesion location and post-ESD bleeding [8–10]. However, one study reported that post-ESD bleeding was significantly less frequent in lower region [11]. In contrast, several other studies have reported that predictors of post-ESD bleeding included middle or lower region [6] or lower region [12]. Similarly, in our study, univariate analysis of 21 bleeding cases showed that there was a significantly higher frequency of bleeding in middle or lower region than in upper region (*P* = 0.036). We suggest that the higher frequency of post-ESD bleeding in middle and lower regions may be due to more active peristalsis in that region, as also suggested by Takizawa et al. [6]. They further suggested that the diameter of the submucosal arteries was larger in the upper region than in the other regions; therefore, intraoperative bleeding occurs more frequently and complete hemostasis is achieved. In contrast, there is a lower frequency of intraoperative bleeding in the middle and lower regions, so that vessels in these regions are not sufficiently coagulated and delayed bleeding may occur. Furthermore, more bile juice reflux in the lower region may contribute to higher frequency of post-ESD bleeding [6]. We agree with this reasoning.

In our study, we recorded and investigated the circumference (anterior wall, posterior wall, lesser curve, or greater curve) in addition to the major axis of the ulcer. Univariate analysis of the 21 bleeding cases showed that bleeding was significantly more frequent in post-ESD ulcers with extended lesser curves (*P* = 0.009). This may be due to the many relatively thick arteries branching from the left gastric artery and the many arteries branching from the right gastric artery forming an arcade with the left gastric artery in a lesser curve [13, 14].

In the present study, the presence of ulcers was the primary predictor of post-ESD bleeding having a significant difference between groups in the multivariate analysis (*P* < 0.005). We suggest that the high frequency of post-ESD bleeding in lesions with ulcers is due to the rich blood vessels in ulcerative regions that grow as part of the healing process of an ulcer [15]. Similarly, vascularization in gastric carcinomas may contribute to rich blood vessels in ulcerative regions. No prior studies have identified this factor as a predictor for post-ESD bleeding. Although the reason for the discrepancy between the previous studies and the present study are unclear, the majority of previous studies had a tendency toward high frequency of post-ESD bleeding in lesions with ulcers [6–8, 10–12]. Several previous studies have reported risk factors for post-ESD bleeding, the results of these studies, including the present study, are diverse, and there is currently no consensus with respect to risk factors. Although the reason for the discrepancy could not be determined, it may include the single-center retrospective study design of each previous studies and various uncertain factors associated with each institution, such as ESD procedures and operators. The lack of consistent results requires further consideration, and meta-analysis of these studies may be useful.

Several measures to control post-ESD bleeding have previously been investigated and reported [6, 16]. Takizawa et al. reported that post-ESD coagulation in the resection area could prevent post-ESD bleeding [6]. The procedure suggested was the procedure we used, coagulation of visible vessels in the resection area using hemostatic forceps when ESD was completed to prevent delayed bleeding. Uedo et al. reported that an endoscopic Doppler ultrasound has possible benefits for prevention of post-ESD bleeding [16]. However, despite these measures, post-ESD bleeding continues to occur regularly.

Commonly, a second-look endoscopy is routinely performed the next day or a few days after ESD before beginning meals to prevent post-ESD bleeding. However, one study reported that a second-look endoscopy after ESD may contribute little to prevention of post-ESD bleeding [7]. If the frequency of post-ESD bleeding was decreased by improved measures to prevent post-ESD bleeding in the future, a second-look endoscopy may become unnecessary.

Furthermore, prospective randomized controlled trials have identified that the use of PPIs results in significantly lower bleeding rates after ESD than the use of H2-receptor antagonists [17, 18]. We also administered a PPI, 20 mg omeprazole, intravenously from the day of ESD to the day before beginning meals.

The present study suggests that treatment of blood vessels on a post-ESD lesion, especially for ulcers, middle or lower regions, or with lesser curves, would contribute to a reduction in the frequency of post-ESD bleeding. In particular, detection of arteries on the ulcer is likely important to prevent serious post-ESD bleeding requiring emergent endoscopic treatment. The findings of the present study and further investigation of post-ESD bleeding may allow establishment of preventive measures for post-ESD bleeding. More effective preventive measures may contribute to reducing the number of days of hospital treatment and the need for follow-up endoscopy.

The limitations of the present study include its single-center retrospective study design and small sample size. Moreover, our data include various uncertain factors, such as operators, instruments, subjects, and periods.

In conclusion, we found that the presence of ulcers is a statistically significant predictor of post-ESD bleeding, and that middle or lower regions and post-ESD ulcers with an extended lesser curve are also considered risk factors.

## Figures and Tables

**Table 1:** Baseline characteristics of patients and gastric lesions.

Characteristic	Patients and gastric lesions (*n* = 161)
Age (yr, mean ± SD)	72.4 ± 8.8
Sex (male/female)	116/45
Anticoagulants/platelets (used/not used)	33/128
Morphology (I/IIa/IIc, IIa + IIc, III)	6/97/58
Histological type (intestinal/diffuse/adenoma)	121/4/36
Depth (M/SM,MP)	145/16
Ulcer findings (presence/absence)	21/140
Location (U/M/L)	22/74/65
Location (U/ML)	22/139
Circumference (AW/GC/LC/PW)	20/45/67/29
Size of resected lesion (mm, mean ± SD)	35.5 ± 11.8
Procedure duration (min, mean ± SD)	81.4 ± 49.4
Number of times hemostatic forceps used (mean ± SD)	4.4 ± 4.8
Post-ESD ulcer (extended LC/not extended LC)	89/72

SD: standard deviation; M: mucosa; SM: submucosa; U: upper third; M: middle third: L, lower third; AW: anterior wall; GC: greater curve; LC: lesser curve; PW: posterior wall; ESD: endoscopic submucosal dissection.

**Table 2:** Univariate analysis of delayed bleeding.

Factor	Present (*n* = 21)	Absent (*n* = 140)	*P* value
Age (yr, mean ± SD)	70.2 ± 11.6	72.8 ± 8.3	0.432
Sex (male/female)	15/6	101/39	0.847
Morphology (I/IIa/IIc, IIa + IIc, III)	1/8/12	5/89/46	0.081
Histological type (intestinal/diffuse/adenoma)	18/1/2	103/3/34	0.267
Depth (M/SM,MP)	21/0	124/16	0.095
Ulcer findings (presence/absence)	9/12	12/128	<0.001*
Location (U/M/L)	0/10/11	22/64/54	0.124
Location (U/ML)	0/21	22/118	0.036*
Circumference (AW/GC/LC/PW)	6/1/11/3	14/44/56/26	0.014*
Size of resected lesion (mm, mean ± SD)	38.9 ± 10.8	35.0 ± 11.9	0.17
Procedure duration (min, mean ± SD)	88.2 ± 33.6	80.4 ± 51.9	0.164
Number of times hemostatic forceps used (mean ± SD)	5.6 ± 3.9	4.3 ± 4.9	0.162
Anticoagulants/platelets (used/not used)	4/17	29/111	0.562
Post-ESD ulcer (extended LC/not extended LC)	17/4	72/68	0.009*

SD: standard deviation; M: mucosa; SM: submucosa; U: upper third; M: middle third; L: lower third; AW: anterior wall; GC: greater curve; LC: lesser curve; PW: posterior wall; ESD: endoscopic submucosal dissection.

*Significantly different.

**Table 3:** Multivariate analysis of delayed bleeding.

Factors		OR	95% CI	*P* value
Morphology	I	1.24	0.11–13.81	0.86
	IIa	0.60	0.18–2.03	0.41
Depth (M/SM,MP)	M	0.24	0.05–1.22	0.09
Ulcer findings	Present	9.73	2.28–41.53	0.002*
Location	ML	1.46	0.26–8.26	0.67
Circumference	AW	1.13	0.23–5.48	0.88
	LC	0.34	0.08–1.47	0.15
	GC	0.11	0.01–1.27	0.08
Size of resected lesion		0.10	0.94–1.05	0.87
Procedure duration		0.99	0.97–1.01	0.17
Post-ESD ulcer	Extended LC	3.23	0.68–15.42	0.14

AW: anterior wall; GC: greater curve; LC: lesser curve; PW: posterior wall; OR: odds ratio; CI: confidence interval.

*Significantly different.

**Table 4:** Analysis of the 21 bleeding cases.

Timing	
(0–2 days after ESD/≥3 days after ESD)	15/6
Bleeding region	
Major axis (U/M/L)	0/10/11
Circumference (AW/GC/LC/PW)	4/0/15/2
At the ulcer (center/margin)	12/9
Nature of the bleeding	
(Spouting/gushing/exposed vessels/oozing)	2/8/5/6
Clinical findings	
(Hematemesis/anemia/shock/transfusion)	6/4/4/2
